# Characterization, antioxidant and antitumor activities of phenolic compounds from *Amomum villosum* Lour.

**DOI:** 10.3389/fnut.2024.1327164

**Published:** 2024-02-06

**Authors:** Ming Zhang, Xi-xiang Shuai, Zhi Wei, Tao-tao Dai, Chang-bin Wei, Ya Li, Jun-jun He, Li-qing Du

**Affiliations:** ^1^South Subtropical Crops Research Institute, China Academy of Tropical Agricultural Sciences, Key Laboratory of Hainan Province for Postharvest Physiology and Technology of Tropical Horticultural Products, Zhanjiang, China; ^2^State Key Laboratory of Food Science and Resources, Nanchang University, Nanchang, China; ^3^Zhanjiang Experimental Station, China Academy of Tropical Agricultural Sciences, Zhanjiang, China

**Keywords:** *Amomum villosum* Lour., phenolic compounds, UPLC-ESI-QTOF-MS/MS, antioxidant, antitumor

## Abstract

*Amomum villosum* Lour. (*A. villosum*), known as Sharen in China, is widely used for culinary and medicinal purposes due to containing a diverse set of bioactive compounds. In this study, the optimum ethanol extraction process was optimized and the composition and biological activities (antioxidant and antitumor) of five different fractions (dichloromethane, petroleum ether, ethyl acetate, *n*-butanol and H_2_O) extracted from the ethanol extract of *A. villosum* were investigated. The results showed that the optimal extraction conditions were extraction temperature 80°C, extraction time 120 min, ethanol concentration 40% and solid–liquid ratio 1:25 g/mL. Moreover, 35 bioactive compounds were successfully identified by UPLC-ESI-QTOF-MS/MS from five factions for the first time, including 12 phenolic acids and derivatives, 2 organic acids, 12 flavonoids and derivatives, 2 oxylipins and 7 proanthocyanidins. Among them, ethyl acetate fraction (Fr-EtOAc) exhibited the highest content of total phenolic (374.01 mg GAE/g DW) and flavonoid (93.11 mg RE/g DW), where vanillic acid, catechin, epicatechin and protocatechuic acid were the predominant phenolic compounds that accounting for 81.65% of the quantified bioactive compounds. In addition, Fr-EtOAc demonstrated excellent total antioxidant activity (IC_50_ of DPPH and ABTS assays were 0.23, 0.08 mg/mL, respectively, and FRAP assay was 322.91 mg VCE/100 g DW) and antitumor activity (1,000 μg/mL, 79.04% inhibition rate). The results could provide guidance for the industrial production and application of *A. villosum.*

## 1 Introduction

*Amomum villosum* Lour. (*A. villosum*), usually called sharen in China, is a member of Zingiberaceae family and is mainly cultivated in Southern China and Southeast Asian countries. The fruit of *A. villosum* was used for medicine purposes could be traced back to the seventh century, and together with *Areca catechu* L., *Morinda officinalis* How. and *Alpinia oxyphylla* Miq. were called “four southern medicines” (1). Nowadays, the fruit of *A. villosum* is also widely used as a spice in culinary due to its non-toxic, aromatic smell and biological activities (2). There is no doubt that the medicinal and edible values of *A. villosum* were related to the chemical ingredients, including volatile oils, non-volatile compounds (phenolics, flavonoids, polysaccharides, et al.) and so on (3).

Currently, lots of research had focused on the volatile oils and polysaccharides from *A. villosum* and their pharmacological effects. As previously reported, Tang et al. (4) found that *A. villosum* volatile oil exhibited antibacterial activity by interfering with the metabolism of methicillin-resistant *Staphylococcus aureus*, and the report described by Liu et al. (5) revealed that *A. villosum* polysaccharides could reduce gastric mucosal injury by promoting the level of reactive oxygen species and inflammatory factors. Although there were several studies on the biological activities of the water extract of *A. villosum*, including absorption characteristics (6), weight loss (7), mitigate hyperlipidemia (8), the composition of phenolic compounds from *A. villosum* was still unclear, which restricted the further utilization of *A. villosum*.

Phenolic compounds have a wide range of bioactive properties, including antioxidant (9, 10), antitumor (11) and other properties. Meanwhile, an increasing number of reports had proved that biological activities were related to the composition of phenolic compounds (12, 13). Therefore, it was very important to take suitable methods for identifying and quantifying the phenolic compounds in plants. Notably, isolation was an essential procedure to enrich the phenolic compounds. And, fractional extraction, as an efficient isolation method, had been utilized to enrich and isolate the target natural compounds by many researchers (13–15). However, there was no report on the systematic study of phenolic compounds from *A. villosum*, such as phenolic compounds composition, antioxidant activities and their correlation.

Based on the aforementioned, this study focused on optimizing the extraction conditions of phenolic compounds in the seeds of *A. villosum* and obtaining five fractions from the ethanol extract by fractional extraction method with five different polarities solvents, and then identifying and quantifying the phenolic compounds composition by UHPLC-ESI-QTOF-MS/MS. Moreover, the antioxidant and antitumor activities were determined and the correlation with phenolic compounds was evaluated by Person correlation analysis. These results could provide guidance for the development and application of *A. villosum*.

## 2 Materials and methods

### 2.1 Materials and reagents

The seeds of *A. villosum* were collected by a five digonal point sampling method from Yangjiang Amomum planting base (Guangdong Province, China) in August 2022. 20 plants without disease and pest were randomly selected when the fruit was easy to separate from stem and crack and the color of seeds became dark brown. The collected samples were fully mixed, packaged with valve bags and labeled, then taken back to the laboratory. 2,2′-azino-bis (3-ethylbenzothiazoline-6-sulfonic acid) (ABTS), 1,1-diphenyl-2-picrylhydrazyl (DPPH), dulbecco’s modified eagle medium (DMEM), fetal bovine serum (FBS), cell counting kit-8 (CCK8), acetonitrile, quercitrin, vanillic acid, gallic acid, syringic acid, protocatechuic acid, catechin, epicatechin, isorhamnetin, rutin, ferulic acid, caffeic acid, hyperoside, isoqercitrin, protocatechualdehyde, quercetin, *p*-coumaric acid and 4-hydroxybenzoic acid were purchased from Sigma-Aldrich Co., Ltd. (Shanghai, China). Petroleum ether, dichloromethane, ethyl acetate, *n*-butanol, sodium carbonate, formic acid and methanol were purchased from Tianjin Fuyu Co., Ltd. (Tianjin, China). All other reagents were analytical grade.

### 2.2 Preparation and extraction of phenolic compounds from *Amomum villosum*

**Figure 1:**
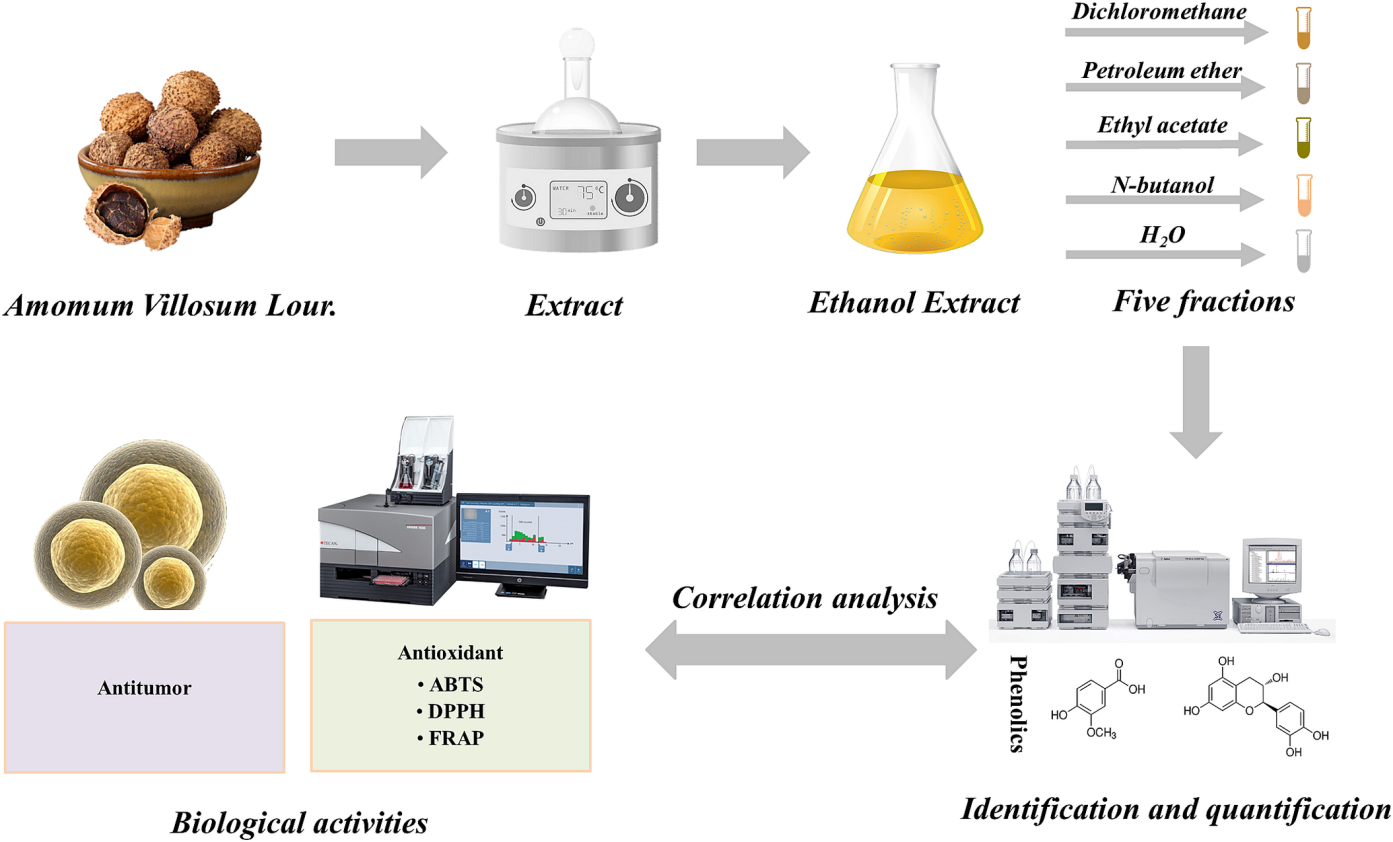
The schematic diagram of the detailed trial protocol of this study.

The seeds of *A. villosum* were washed with distilled water and then placed into a hot air dryer (101-A1, Wuxi marit Co., Ltd., Jiangsu, China) at 50°C until constant weight. The dried seeds were ground to powder (particle size less than 50 μm) using a high-speed grinder (HR-10, Zhejiang harui Co. Ltd., Zhejiang, China). The *A. villosum* powder was taken into extraction vessel and mixed with a defined solid-to-solvent ratio of ethanol solution, and then extracted at the set temperature and time according to the experimental design (Table 1). After extraction, the extract was separated from the solid by centrifuging at 4500 rpm for 15 min, and the supernatant was collected and concentrated to approximate 50 mL at 50°C under vacuum by a rotary evaporator (RE-2000A, Shanghai yarong Co. Ltd., Shanghai). The concentrated solution was diluted to a total volume of 100 mL with distilled water, and successively extracted using petroleum ether, dichloromethane, ethyl acetate and *n*-butanol at a ratio of 1:1 (v/v), 3 times. And the extract solutions were condensed to 5 mL, and the organic phase and H_2_O phase extracts were dried by nitrogen blowing concentrator and lyophilizer, respectively. Finally, there were five fractions were prepared from the ethanol extract of *A. villosum*, including petroleum ether fraction (Fr-PE, 0.47%), dichloromethane fraction (Fr-CH_2_Cl_2_, 0.95%), ethyl acetate fraction (Fr-EtOAc, 0.46%), *n*-butanol fraction (Fr-nBuOH, 1.95%) and H_2_O fraction (Fr-H_2_O, 10.30%), and the yields were shown in Table 2. The dried samples were collected and stored at-18°C (in a dark condition) for further investigations. A schematic representation of the experiments carried out in this study was shown in Figure 1.

**Table 1:** The design of orthogonal test (L_9_ (3^4^)) and range analysis.

Number	Factors				TPC(mg GAE/g DW)
	Extraction temperature (°C)	Extraction time (min)	Ethanol concentration (%)	Solid–liquid ratio (g/mL)	
1	60	90	40	1:20	15.85 ± 0.11
2	60	120	50	1:25	15.94 ± 0.12
3	60	150	60	1:30	13.75 ± 0.67
4	70	90	50	1:30	17.40 ± 0.07
5	70	120	60	1:20	15.42 ± 0.45
6	70	150	40	1:25	18.37 ± 0.28
7	80	90	60	1:25	16.80 ± 0.14
8	80	120	40	1:30	19.18 ± 0.08
9	80	150	50	1:20	17.83 ± 0.49
k_1_	15.18	16.68	17.80	16.37	
k_2_	17.06	16.85	17.06	17.04	
k_3_	17.94	16.65	15.32	16.78	
**Range**	**2.76**	**0.20**	**2.47**	**0.67**	

TPC, total phenolic content; GAE, gallic acid equivalent; DW, dried weight.

**Table 2:** The yields of different fractions.

Fractions	Fr-PE	Fr-CH_2_Cl_2_	Fr-EtOAc	Fr-nBuOH	Fr-H_2_O
Yields (%)	0.47 ± 0.02	0.95 ± 0.03	0.46 ± 0.02	1.95 ± 0.13	10.30 ± 0.21

Fr-PE, petroleum ether fraction; Fr-CH_2_Cl_2_, dichloromethane fraction; Fr-EtOAc, ethyl acetate fraction; Fr-nBuOH, n-butanol fraction; Fr-H_2_O, H_2_O fraction.

### 2.3 Determination of total phenolic content and total flavonoid content

The TPC and TFC of five fractions from *A. villosum* were evaluated by the Folin–Ciocalteu method (16) and aluminum chloride colorimetric method (17), respectively. The absorbance values of TPC and TFC assays were read at 750 nm and 510 nm by a UV–Vis spectrophotometer (U-T1810, Beijing puxi Co., Ltd., Beijing, China), respectively. Finally, the results of TPC were quantified as mg gallic acid equivalent (GAE) per gram of dried weight (DW) of five fractions (mg GAE/g DW), while the results of TFC were expressed as mg rutin equivalent (RE) per gram of DW of five fractions (mg RE/g DW).

### 2.4 Antioxidant activities

#### 2.4.1 DPPḤ and ABTS^+^ free radical scavenging capacities

The DPPḤ and ABTS^+^ free radical scavenging assays were performed according to the previous reports by Zhou et al. (18) and da Costa et al. (19), respectively. The absorbance values of DPPḤ and ABTS^+^ free radical scavenging assays were measured at 517 nm and 734 nm using a microplate reader (Bio Tek, United States), respectively. Finally, the DPPḤ or ABTS^+^ free radical scavenging rate was calculated by the following Equation (1).


(1) DPPH·or ABTS+free radical scavenging rate%=1−A0/A1×100

where A_0_ and A_1_ refer to the absorbance values of the sample and control, respectively. Finally, the DPPḤ and ABTS^+^ free radical scavenging capacities denoted as IC_50_, represented the concentration of the sample when the DPPḤ or ABTS^+^ free radical scavenging rate was 50% which was calculated by the fitting curves.

#### 2.4.2 The ferric ion reducing antioxidant power

The FRAP assay was measured according to the method reported by Pourshoaib et al. (20). The absorbance value was measured at 593 nm and the results of FRAP were expressed as mg vitamin C equivalent (VCE) per 100 grams of DW of five fractions (mg VCE/100 g DW).

### 2.5 Cytotoxicity and antitumor activity

To determine the cytotoxicity and antitumor activity of five fractions of *A. villosum*, the human umbilical vein endothelial (HUVE) cells (Shanghai Meixuan Biotechnology Co., Ltd., Shanghai, China) and HeLa cell lines (Beyotime Institute of Biotechnology, Shanghai, China) were used, respectively. The cells were cultured in DMEM basic medium supplemented with 10% (v/v) FBS and 1% (v/v) penicillin–streptomycin at 37°C in an atmosphere with 5% CO_2_ (21). When the cell confluence reached ~70%, the cells were then further used for the cytotoxicity and antitumor activity assays. In this study, the cytotoxicity and antitumor activity were analyzed by CCK8 assays based on the method described by Pu et al. (22) with slightly modifications. Briefly, the cells were seeded into 96-well plates at a density of 5 × 10^4^ cells/well and allowed to grow at 37°C for 12 h. For determination of the cytotoxicity of five fractions, 10 μL of 100, 500, 1,000 μg/mL of five fractions were added into the wells, respectively, and incubated at 37°C for 48 h followed by 10 μL of CCK8 reagent for 2 h. Subsequently, the absorbance value was measured at 450 nm using a microplate reader (Multiskan FC, Thermo Fisher Scientific, Waltham, MA, United States). For determination of the antitumor activity of five fractions, 100, 200, 400, 600, 800, and 1,000 μg/mL of five fractions were evaluated according to the procedure of cytotoxicity assay. Finally, the results of the cytotoxicity and antitumor activity of five fractions were expressed by cell viability (%) and inhibition rate (%), respectively.

### 2.6 UHPLC-ESI-QTOF-MS/MS analysis of phenolic compounds

The phenolic compounds in five fractions were identified using a Ultra-High Performance Liquid Chromatography (UHPLC; 1,290 Infinity, Agilent, United States) equipped with ZORBAX Eclipse Plus C18 (100 mm × 2.1 mm, 1.8 μm, Agilent Technologies, Santa Clara, CA) column and Triple TOF™ 5600^+^ electrospray time-of-flight high resolution mass (AB Sciex, Foster City, CA, United States). The gradient elution of the mobile phase was conducted using solvent A (water) and B (acetonitrile), respectively, and the process was as follows: 0 min, 99% B; 4 min, 95% B; 23 min, 45% B, 26 min, 10% B, 27 min, 10% B, 28 min, 99% B and hold it for 4 min, flow rate = 0.3 mL/min, injection volume = 10 μL, column temperature = 40°C. The UHPLC system was coupled to a quadrupole-time-of-flight orthogonal accelerated Q-TOF mass spectrometer equipped with an electrospray ionization source (ESI) and operating parameters as described by Wang et al. (23). Finally, the composition of phenolic compounds from *A. villosum* was analyzed by matching the database. The standard compounds, calibration lines and method validations for quantification of phenolic compounds from *A. villosum* were presented in Table 3. The content of phenolic compounds was expressed as mg of phenolic compounds per gram of DW of five fractions (mg/g DW).

**Table 3:** Contents of 17 major phenolic compounds in five *A. villosum* fractions (mg/g DW).

Number	Compounds	Regression equation	*R*^2^	Fr-PE	Fr-CH_2_Cl_2_	Fr-EtOAc	Fr-nBuOH	Fr-H_2_O
1	Quercitrin	y = 1E+06x − 16740	0.9997	ND	ND	15.61 ± 0.58	0.81 ± 0.03	ND
2	Vanillic acid	y = 47,194x + 181.1	0.9993	ND	2.38 ± 0.10	93.70 ± 3.13	0.89 ± 0.07	ND
3	Gallic acid	y = 362,825x − 12734	0.9959	ND	ND	0.39 ± 0.00	ND	ND
4	Syringic acid	y = 219,826x − 3786.1	0.9995	ND	ND	3.36 ± 0.01	ND	ND
5	Protocatechuic acid	y = 657,872x − 7827.4	0.9999	ND	ND	38.93 ± 1.10	1.35 ± 0.01	0.27 ± 0.01
6	(+/−) Catechin	y = 589,073x − 6535.7	0.9995	ND	ND	72.67 ± 0.48	4.10 ± 0.08	ND
7	Epicatechin	y = 879,555x − 17623	0.9982	ND	ND	69.87 ± 1.75	5.61 ± 0.22	ND
8	Isorhamnetin	y = 1E+06x + 57,244	0.9818	ND	ND	2.64 ± 0.03	ND	ND
9	Rutin	y = 2E+06x − 62151	0.9987	ND	ND	0.43 ± 0.00	0.68 ± 0.03	ND
10	Ferulic acid	y = 515,351x − 8331.6	0.9991	ND	0.59 ± 0.02	2.52 ± 0.03	ND	ND
11	Caffeic acid	y = 3E+06x − 60142	0.9987	ND	ND	0.49 ± 0.02	ND	ND
12	Hyperoside	y = 2E+06x − 29401	0.9997	ND	ND	2.24 ± 0.18	0.56 ± 0.04	ND
13	Isoqercitrin	y = 5E+06x − 55014	0.9988	ND	ND	2.20 ± 0.60	0.81 ± 0.06	ND
14	Protocatechualdehyde	y = 3E+06x + 51,312	0.9924	ND	0.21 ± 0.01	4.21 ± 0.04	0.04 ± 0.00	0.04 ± 0.00
15	Quercetin	y = 2E+06x − 63871	0.9936	ND	ND	18.40 ± 0.45	ND	ND
16	*p*-coumaric acid	y = 1E+06x − 82666	0.9985	ND	ND	3.61 ± 0.05	ND	ND
17	4-hydroxybenzoic acid	Y = 1E+06x − 14490	0.9992	ND	ND	5.76 ± 0.26	ND	ND
Total				0	3.18	337.03	14.85	0.31

DW, dried weight; Fr-PE, petroleum ether fraction; Fr-CH_2_Cl_2_, dichloromethane fraction; Fr-EtOAc, ethyl acetate fraction; Fr-nBuOH, n-butanol fraction; Fr-H_2_O, H_2_O fraction.

### 2.7 Statistical analyses

All experiments were conducted in triplicate and all results were shown as mean value ± standard deviation. The analysis of significant differences was performed at *p* = 0.05 by Duncan’s tests through SPSS Statistic 26.0 (IBM software, United States). The nonlinear polynomial fit of antioxidant activities results and graph drawing were conducted by Origin 9.0 (Origin Lab Co., Northampton, MA, United States).

## 3 Results and discussion

### 3.1 The extraction of phenolic compounds from *Amomum villosum*

**Figure 2:**
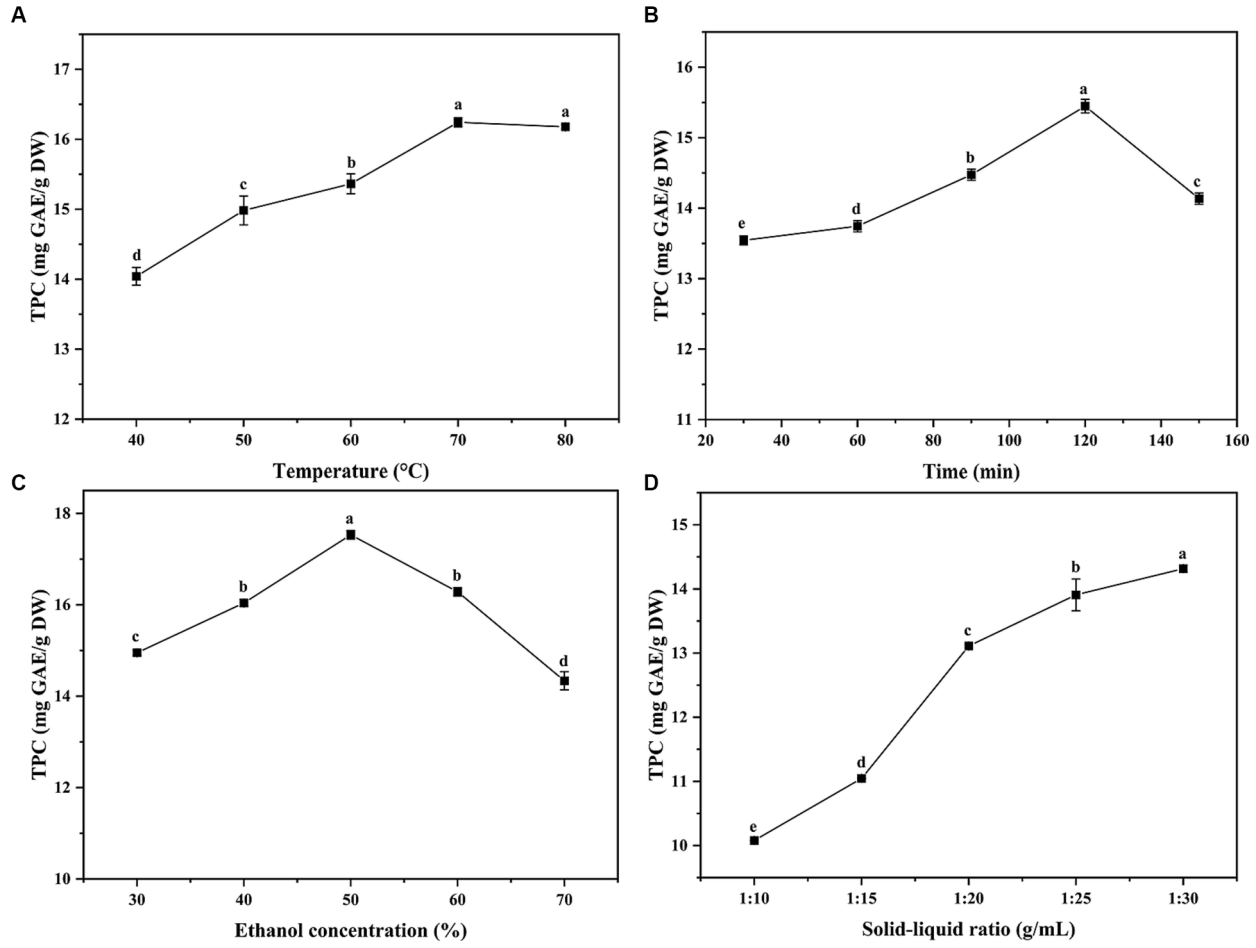
The effects of temperature, time, ethanol concentration and solid-liquid ratio on total phenolic content (TPC). GAE: gallic acid equivalent, DW: dried weight. Superscript letters in each point indicated significant differences between different letters (*p* < 0.05).

The yield of phenolic compounds was affected by many extraction factors, such as extraction time, solvent type and temperature, etc. The single-factor experiments in this study were performed at various solid–liquid ratio (g/mL), ethanol concentration (%), temperature (°C) and time (min). Figure 2 presented the results of four single-factor experiments, and it showed that the TPC value of ethanol concentration, temperature and time experiments exhibited a tendency of increasing first and then decreasing, while the solid–liquid ratio experiment showed an increasing trend first and then stabilizing. Considering the cost, energy consumption and other factors, the optimal extraction conditions for single-factor were obtained from the response of TPC to extraction parameters: extraction temperature of 70°C, extraction time of 120 min, ethanol concentration of 50% and solid–liquid ratio of 1:30 g/mL, respectively.

Based on the above results, an orthogonal test (L_9_ (3^4^)) was employed to optimize the extraction conditions of phenolic compounds in the seeds of *A. villosum*, and the results were listed in Table 1. It was shown that the TPC values were in a range of 13.75 ± 0.67 mg GAE/g DW to 19.18 ± 0.08 GAE/g DW. Meanwhile, the influence order of four factors was obtained from the range of Table 1 and the F ratio of Table 4: extraction temperature > ethanol concentration > solid–liquid ratio > extraction time. In general, the extraction temperature was related to the mass transfer rate and the TPC increased within a certain temperature range, and the extraction time showed a positive response of TPC within a certain time range. While the excessive solid–liquid rate and ethanol concentration led to more energy consumption. Therefore, considering the k_i_ (i = 1, 2, 3) values from Table 1 and the effect of factors, the optimum extraction conditions of phenolic compounds from *A. villosum* were as follows: extraction temperature of 80°C, extraction time of 120 min, ethanol concentration of 40% and solid–liquid ratio of 1:25 g/mL. Meanwhile, the TPC value was 19.65 ± 0.13 mg GAE/g DW under the above-optimized extraction conditions, which was 0.47 mg GAE/g DW higher than the highest TPC value (19.18 mg GAE/g DW) of orthogonal test, suggesting that the extraction conditions optimized by orthogonal test were credible.

**Table 4:** The variance analysis of orthogonal test.

Factors	Sum of squared deviations	Degree of freedom	F ratio
Extract temperature (°C)	11.909	2	2.131
Extraction time (min)	0.066	2	0.012
Ethanol concentration (%)	9.691	2	1.734
Solid–liquid ratio (g/mL)	0.685	2	0.123
Error	22.35	8	

### 3.2 TPC and TFC

**Figure 3:**
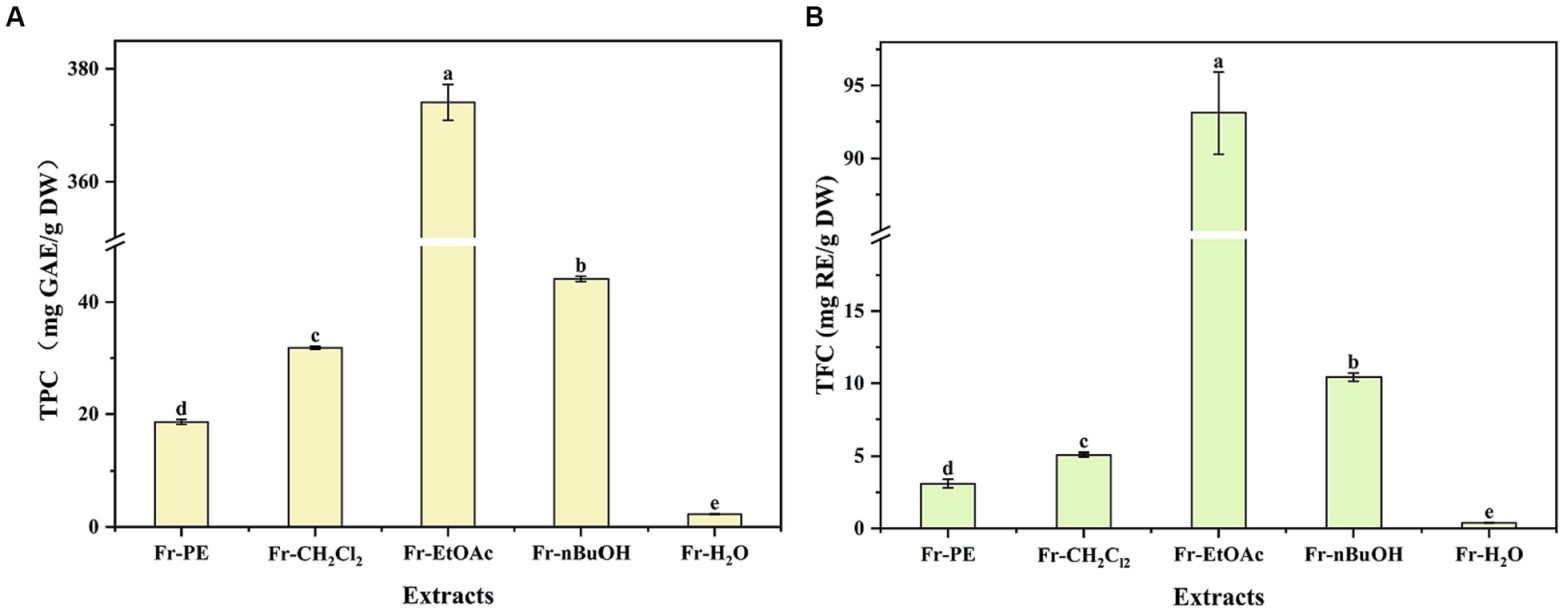
The effects of extraction methods on TPC and TFC of *A. villosum* TPC: total phenolic content, TFC: total flavonoid content, GAE: gallic acid equivalent, RE: rutin equivalent, DW: dried weight, Fr-PE: petroleum ether fraction, Fr-CH_2_Cl_2_: dichloromethane fraction, Fr-EtOAc: ethyl acetate fraction, Fr-nBuOH: *n*-butanol fraction, Fr-H_2_O: H_2_O fraction. Superscript letters in each column indicated significant differences between different letters (*p* < 0.05).

Phenolic compounds, as the plant secondary metabolites, their contents are commonly considered to be responsible for biological activities, including antioxidant, antitumor and antibacterial (24, 25). As presented in Figure 3, TPC and TFC values ranged from 2.29 to 374.01 mg GAE/g DW and 0.38 to 93.11 mg RE/g DW, respectively, and significant differences were observed in TPC and TFC values of five fractions (*p* < 0.05). Among five fractions, the highest TPC and TFC values were found in Fr-EtOAc, while Fr-H_2_O displayed the lowest, which were consistent with the studies conducted by Fan et al. (13) and Bhardwaj et al. (26) that solvents with too high or too low polarity showed low response for TPC and TFC. Meanwhile, the results were also in line with the fact that water was not effective for the extraction of phenolic compounds (15). Therefore, the higher TPC and TFC values of Fr-EtOAc indicated that extraction solvents with medium polarity were more effective for the extraction of phenolic compounds from *A. villosum*. However, EtOAc presented a low response for TPC in the study of Dias et al. (27) who evaluated the extraction ability of different solvents for bioactive compounds from *C. baccatum* fruit, which confirmed the assumption of Herrera-Pool et al. (14) that different species of plants and extraction conditions of phenolic compounds related to the different responses for TPC.

### 3.3 Identification of phenolic compounds from *Amomum villosum*

**Figure 4:**
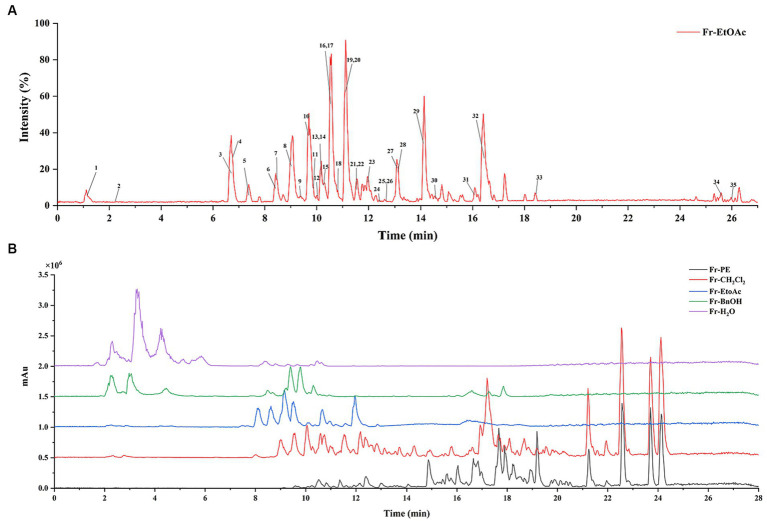
**(A)** Represented the UHPLC-ESI-QTOF-MS/MS base peak chromatogram for *A. villosum* extracts. **(B)** Denoted the DAD spectra of five fractions.

In this study, the phenolic compounds in five fractions of *A. villosum* were analyzed by UHPLC-ESI-QTOF-MS/MS according to the method illustrated in Section 2.6. As shown in Table 5 and Figure 4, a total of 35 bioactive compounds in five fractions of *A. villosum* were successfully verified based on the retention time (RT), fragmentation pattern of MS and MS^2^ mass spectra, including 12 phenolic acids and derivatives, 2 organic acids, 12 flavonoids and derivatives, 2 oxylipins and 7 proanthocyanidins.

**Table 5:** The chemical composition identified in *A. villosum* extracts based on the UPLC-ESI-QTOF-MS/MS analysis.

No.	Name	Rt(min)	Molecular	Ion	Measured (m/z)	MS^2^ ion fragment
1	Malic acid	1.335	C_4_H_6_O_5_	[M-H]^−^	133.0151	115, 71
2	Citric acid	2.262	C_6_H_8_O_7_	[M-H]^−^	191.0208	111, 87
3	Protocatechuic acid	6.655	C_7_H_6_O_4_	[M-H]^−^	153.0198	109, 91
4	Gallic acid	6.676	C_7_H_6_O_5_	[M-H]^−^	169.0144	151, 125, 107, 83
5	Vanillic acid hexoside isomer	7.609	C_14_H_18_O_9_	[M-H]^−^	329.0898	269, 209, 167, 123
6	4-hydroxybenzoic acid	8.384	C_7_H_6_O_3_	[M-H]^−^	137.0253	93, 65
7	Protocatechualdehyde	8.408	C_7_H_6_O_3_	[M-H]^−^	137.0250	119, 108, 91
8	Procyanidin B isomer	9.025	C_30_H_26_O_12_	[M-H]^−^	577.1422	425, 407, 289, 245
9	Catechin hexoside isomer	9.530	C_21_H_24_O_11_	[M-H]^−^	451.1250	361, 331, 289, 245
10	(+/−) Catechin	9.681	C_15_H_14_O_6_	[M-H]^−^	289.0745	245, 203, 151, 123, 109
11	Vanillic acid hexoside isomer	9.941	C_14_H_18_O_9_	[M-H]^−^	329.0898	269, 209, 167, 123
12	Catechin hexoside isomer	10.033	C_21_H_24_O_11_	[M-H]^−^	451.1250	361, 331, 289, 245
13	Vanillic acid	10.091	C_8_H_8_O_4_	[M-H]^−^	167.0348	152, 123, 108, 91
14	Procyanidin C isomer	10.271	C_45_H_38_O_18_	[M-H]^−^	865.2126	695, 577, 425, 407, 289
15	Caffeic acid	10.343	C_9_H_8_O_4_	[M-H]^−^	179.0356	135, 117, 107
16	Procyanidin B isomer	10.483	C_30_H_26_O_12_	[M-H]^−^	577.1402	425, 407, 289, 245
17	Procyanidin D	10.523	C_60_H_50_O_24_	[M-H]^−^	1153.2767	865, 575, 407, 287
18	Syringic acid	10.821	C_9_H_10_O_5_	[M-H]^−^	197.0458	182, 167, 153, 123, 95
19	Epicatechin	11.098	C_15_H_14_O_6_	[M-H]^−^	289.0745	245, 203, 151, 123, 109
20	Procyanidin C isomer	11.544	C_45_H_38_O_18_	[M-H]^−^	865.2103	695, 577, 425, 407, 289
21	Catechin pentoside	11.559	C_21_H_23_O_10_	[M-H]^−^	435.1315	361, 331, 289, 245, 151
22	*p*-coumaric acid	12.095	C_9_H_8_O_3_	[M-H]^−^	163.0403	119, 93
23	Procyanidin A	12.589	C_30_H_24_O_12_	[M-H]^−^	575.1252	449, 289, 285, 245
24	Rutin	12.908	C_27_H_30_O_16_	[M-H]^−^	609.1489	301, 271, 255, 179, 151
25	Ferulic acid	12.92	C_10_H_10_O_4_	[M-H]^−^	193.0516	178, 149, 133, 121
26	Procyanidin B isomer	13.073	C_30_H_26_O_12_	[M-H]^−^	577.1405	425, 407, 289, 245
27	Isoqercitrin	13.107	C_21_H_20_O_12_	[M-H]^−^	463.0903	301, 271, 243, 151
28	Hyperoside	13.244	C_21_H_20_O_12_	[M-H]^−^	463.0903	301, 271, 255, 243, 151
29	Quercitrin	14.106	C_21_H_20_O_11_	[M-H]^−^	447.0974	301, 271, 255, 243, 151
30	Syringaldehyde	14.674	C_9_H_10_O_4_	[M-H]^−^	181.0510	166, 151, 123
31	Catechin glucuronide	16.193	C_22_H_26_O_11_	[M-H]^−^	465.1242	421, 341, 289
32	Quercetin	16.394	C_15_H_10_O_7_	[M-H]^−^	301.0380	273, 245, 179, 151, 121
33	Isorhamnetin	18.419	C_16_H_12_O_7_	[M-H]^−^	315.0539	271, 227, 163, 151, 107
34	9-Hydroxy-10,12,15-octadecatrienoic acid	25.47	C_18_H_30_O_3_	[M-H]^−^	293.2129	275, 183, 171
35	9-Hydroxy-10,12-octadecatrienoic acid	26.064	C_18_H_32_O_3_	[M-H]^−^	295.2298	277, 195, 171

#### 3.3.1 Organic acids

As shown in Figure 4A, peaks 1 and 2 presented characteristic MS^2^ fragment ions at m/z 115 and 111, respectively, resulting from the loss of a water molecule from malic acid and the absence of a water molecule as well as a CO_2_ group from citric acid, which had been verified by Oldoni et al. (28). These compounds had also been reported in apple (29), grape (30) and *Elaeagnus angustifolia* L. flower (31). It is worth noting that malic acid and citric acid are the important intermediates of the tricarboxylic acid cycle, which participate in regulating the metabolism of sugars, fatty acids and amino acids (32). Additionally, the content of organic acids can also be responsible for the organoleptic character, storage and preservation of food.

#### 3.3.2 Phenolic acids and its derivatives

##### 3.3.2.1 Hydroxybenzoic acids

Peaks 3, 4, 6, 13, and 18 showed precursor fragment peaks at m/z 153.0198, 169.0144, 137.0253, 167.0348 and 197.0458 [M-H]^−^, respectively, and characteristic MS^2^ fragment ions at m/z 109, 125, 93, 123 and 153 due to the loss of CO_2_ [M-H-44]^−^. Compared with the previous literatures, peaks 3, 4 and 6 were identified as protocatechuic acid (33), gallic acid (28) and 4-hydroxybenzoic acid (34). In addition, peak 13 also presented characteristic MS^2^ fragment ions at m/z 152 [M-H-CH_3_]^−^ and 108 [M-H-CO_2_-CH_3_]^−^, suggesting peak 13 was vanillic acid according to a previous report in ribes stenocarpum by Jiang et al. (33). The MS^2^ fragment ions of peak 18 further showed characteristic MS^2^ fragment ions at m/z 182 and 166, resulting from the loss of- CH_3_ (15) and -OCH_3_ (31) in negative ion mode, respectively, in the structures, which presented a characteristic fragmentation behavior of syringic acid (35).

##### 3.3.2.2 Hydroxycinnamic acids

Peak 22 (RT = 12.095 min) showed a deprotonated molecule ion at m/z 163.0403 [M-H]^−^, which produced a MS^2^ fragment ion at m/z 119 [M-H-CO_2_]^−^, corresponding to the *p*-coumaric acid as the report by Ali et al. (36). Furthermore, it could be observed that peak 15 presented a characteristic MS^2^ fragment ion at m/z 135 that indicated the addition of a oxygen atom compared with peak 22, suggesting peak 15 was caffeic acid, which was agreed with the characteristic fragment ions of caffeic acid in adlay bran (34). Moreover, it was inferred that peak 25 was formed by the introduction of -OCH_3_ into the benzene ring of peak 15 compared with the molecular weight. And peak 25 was further identified based on the MS^2^ fragment ions at m/z 178 and 149, suggesting the loss of -CH_3_ and CO_2_ from the precursor ion at m/z 193.0403 [M-H]^−^ (36), respectively. Thus, peak 25 was identified as ferulic acid.

##### 3.3.2.3 Other phenolic acids derivatives

Peaks 5 (RT = 7.609 min) and 11 (RT = 9.941 min) exhibited the same precursor ion [M-H]^−^ at m/z 329.0898 with the same MS^2^ fragment ions at m/z 269, 209, 167, 123, suggesting that peaks 5 and 11 were structural isomers. It also could be observed a characteristic MS^2^ fragment ion at m/z 167 originated from the loss of glucose (162 Da), which corresponded to a glucose linked to a vanillic acid moiety (33). Therefore, peaks 5 and 11 were identified as vanillic acid hexoside isomer. Peak 7 was tentatively identified as protocatechualdehyde via its MS^2^ fragment ion at m/z 108, resulting from the loss of -CHO from parent ion 137.0250 [M-H]^−^ (37). Peak 30 was characterized as syringaldehyde due to two characteristic MS^2^ fragment ions at m/z 166 and 151, implying the loss of one or two methyl groups (38).

#### 3.3.3 Flavonoids and its derivatives

##### 3.3.3.1 Flavonols

Peaks 24, 27, and 29 exhibited deprotonated molecule ions at m/z 609.1489, 463.0903 and 447.0974 [M-H]^−^, which produced the same MS^2^ fragment ion at m/z 301 (C_15_H_9_O_7_, the same with that of peak 32), indicating the loss of a rutinosyl disaccharide moiety (162 + 146 Da), glucoside group (162 Da) and rhamnose residue (146 Da), respectively. Therefore, peaks 24, 27, 29, and 32 could be easily identified as rutin, isoqercitrin, quercitrin and quercetin, respectively, which corresponded to the previous research (39, 40). According to the report by Zhong et al. (41), peak 28 was characterized as hyperoside due to the same deprotonated ion with peak 27 at m/z 463.0903 [M-H]^−^ and a characteristic MS^2^ fragment ion at m/z 255. Zhou et al. (42) suggested deprotonated ion at m/z 315.0539 [M-H]^−^ and MS^2^ fragment ion at m/z 151 as characteristic fragment ions in the identification of isorhamnetin in *ginkgo biloba* fallen leaves, providing the tentative analysis of peak 33.

##### 3.3.3.2 Flavanols

Peaks 10 and 19 presented the same parent ion at m/z 289.0745 [M-H]^−^ with different retention times, which gave MS^2^ fragment ions at m/z 245 [M-H-CO_2_]^−^ and 109 [M-H-C_9_H_8_O_4_]^−^, indicating that both peaks were isomers, corresponding to catechin and epicatechin as report by Liu et al. (43) who investigated the phenolic compounds in the internal fruit septum of walnuts.

##### 3.3.3.3 Other flavonoid derivatives

Peaks 9 and 12 exhibited the same deprotonated molecule ion at m/z 451.1250 [M-H]^−^ and the entire same MS^2^ fragment ions at m/z 361, 331, 289 and 245, leading to the fact that peaks 9 and 12 were isomers. Furthermore, the characteristic MS^2^ fragment ions at m/z 245 and 109, suggesting the loss of CO_2_ and C_9_H_8_O_4_, respectively from parent ion, indicating peaks 9 and 12 were catechin and epicatechin (43). Peak 21 was characterized as a catechin-pentoside isomer, owing to the precursor ion at m/z 435.1315 [M-H]^−^ (C_21_H_23_O_10_), and MS^2^ fragment ions at m/z 331 and 289, indicating the addition of a pentose to catechin (44). Peak 31, with parent ion at m/z 465.1242 [M-H]^−^, was identified as catechin glucuronide, and it was further confirmed by the MS^2^ fragment ion at m/z 421 [M-H-CO_2_]^−^ and 289 (catechin) (44).

#### 3.3.4 Procyanidins

Procyanidins are formed by the polymerization of different amounts of catechin or epicatechin. Obviously, the fragment ions of procyanidins contain the parent ions of catechin or epicatechin. In this study, peak 23 was speculated to be procyanidin A, owing to the precursor ion at m/z 575.1252 [M-H]^−^ (C_30_H_24_O_12_), and MS^2^ fragment ion at m/z 289 assigned to catechin (45). Moreover, peaks 8, 16 and 26 exhibited the same parent ion at m/z 577.1349 [M-H]^−^ (C_30_H_26_O_12_) and base peak fragment ions at m/z 425, 407, 289 and 245, suggesting that the three peaks should be isomers of procyanidin B (9), respectively. Peaks 14 and 20 were characterized as procyanidin C due to the parent ion at m/z 865.2103 [M-H]^−^ presented a characteristic fragmentation behavior of procyanidin C at m/z 695, 577, 425, 407 and 289 (43). Piątczak et al. (46) suggested that parent ion at m/z 1153.2767 [M-H]^−^ and the characteristic MS^2^ fragment ions at m/z 865, 575 and 287 demonstrated the existence of monomer dimeric and trimeric of catechin and its multiple, respectively. Thus, peak 17 was identified as procyanidin D.

#### 3.3.5 Oxylipins

Peak 34 presented a precursor ion at 293.2129 [M-H]^−^ and MS^2^ fragment ions at 275, 183 (C_11_H_19_O_2_) and 171 (C_9_H_16_O_3_), indicating the breakage occurred at C9-C10 bond, and suggesting the absence of a C=C bond at C1-C9 and the presence of two or three C=C bonds on C10-C18. And then it was speculated as 9-hydroxy-10,12,15-octadecatrienoic acid (47). Analogously, peak 35 (295.2298 [M-H]^−^, C_18_H_32_O_3_) with diagnostic MS^2^ fragment ions at m/z 277,195 and 171 and was proposed as 9-hydroxy-10,12-octadecadienoic acid (47).

### 3.4 Quantification of phenolic compounds in five *Amomum villosum* fractions

In the present work, phenolic compounds in five *A. villosum* fractions were quantified using an UHPLC-ESI-QTOF-MS/MS. As shown in Figure 4B, a total of 17 major phenolic compounds from *A. villosum* were successfully quantified, including quercitrin, vanillic acid, gallic acid, syringic acid, protocatechuic acid, catechin, epicatechin, isorhamnetin, rutin, ferulic acid, caffeic acid, hyperoside, isoquercitrin, protocatechualdehyde, quercetin, *p*-coumaric acid and 4-hydroxybenzoic acid, which was very essential to identify the phenolic compounds composition for the further utilization of *A. villosum*.

As depicted in Table 3, the highest phenolic compound content was found in Fr-EtOAc (337.03 mg/g DW), followed by Fr-nBuOH (14.85 mg/g DW), Fr-CH_2_Cl_2_ (3.18 mg/g DW), Fr-H_2_O (0.31 mg/g DW) and Fr-PE, which were consistent with the results of TPC and TFC (Figure 3). Interestingly, Fr-EtOAc contained all of the 17 quantified phenolic compounds, while only 9, 3 and 2 kinds of the quantified phenolic compounds were found in Fr-nBuOH, Fr-CH_2_Cl_2_ and Fr-H_2_O, respectively. Interestingly, Fr-EtOAc contained all of the 17 quantified phenolic compounds, while only 9, 3 and 2 kinds of the quantified phenolic compounds were found in Fr-nBuOH, Fr-CH_2_Cl_2_ and Fr-H_2_O, respectively. Strangely, among the 17 quantified phenolic compounds, no phenolic compounds were determined in Fr-PE. On one hand, low TPC and TFC of Fr-PE (Figure 3) were responsible for the results. On the other hand, it might be due to the polarity difference between quantified phenolic compounds and the mobile phase of UHPLC, resulting in the poor solubility of target phenolic compounds in the mobile phase.

For individual phenolic compounds, mostly individual phenolic compound contents (except rutin) of Fr-EtOAc were higher than the other four fractions. Meanwhile, the predominant phenolic compounds in Fr-EtOAc were vanillic acid (93.70 mg/g DW), catechin (72.67 mg/g DW), epicatechin (69.87 mg/g DW) and protocatechuic acid (38.93 mg/g DW), accounting for 81.65% of 17 phenolic compounds. In other words, Fr-EtOAc was rich in phenolic compounds with hydroxybenzoic acids and flavonols as major phenolic compounds. Similar results were also found in the report by Fan et al. (13) who found that catechin and epicatechin were the predominant phenolic compounds in Fr-EtOAc of *A. tsaoko*. Although some reports confirmed that the water extract of *A. villosum* had many biological activities (7, 8), there was not much research on the composition of phenolic compounds from *A. villosum*. And this is the first time to report the composition of phenolic compounds from the seeds of *A. villosum*. Additionally, the quantitative results confirmed that Fr-EtOAc was the best fraction for the further utilization of phenolic compounds from *A. villosum*.

### 3.5 Antioxidant activities

It was well known that each evaluation method of antioxidant activity has its particularities. Therefore, the ABTS, DPPH and FRAP assays (48, 49) were used to evaluate the antioxidant activities of phenolic compounds in five fractions of *A. villosum*. It was noted that the results of ABTS and DPPH assays were expressed by IC_50_ values in this study, which represented the concentration of phenolic compounds when the scavenging rate of ABTS^+^ or DPPḤ free radicals was 50%. Thus, the lower IC_50_ value indicates stronger free radicals scavenging capacities and antioxidant activities. Meanwhile, the VCE was used to express the FRAP value, which was positively correlated with the antioxidant activities.

**Figure 5:**
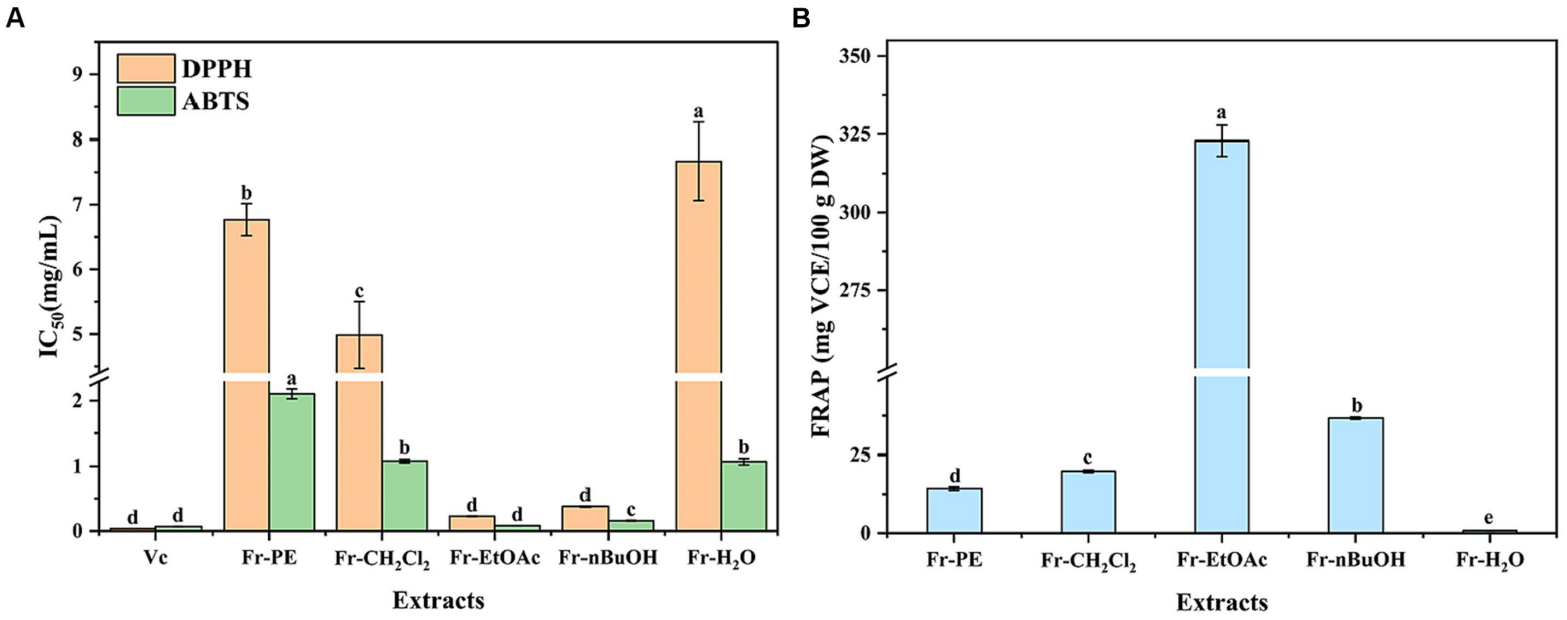
The effects of extraction methods on antioxidant activities of five fractions (Fr-PE: petroleum ether fraction, Fr-CH_2_Cl_2_: dichloromethane fraction, Fr-EtOAc, ethyl acetate fraction, Fr-nBuOH, *n*-butanol fraction, Fr-H_2_O, H_2_O: H_2_O fraction) of *A. villosum*, including 1,1-Dipheyl-2-picryl-hydrazyl (DPPH) and 2,2′-azinobis (3-ethylbenzothiazoline-6-sulphonic acid) (ABTS) free radical scavenging capacity and ferric ion reducing antioxidant power (FRAP). VCE, vitamin C equivalent, DW: dried weight. Superscript letters in each column indicated significant differences between different letters (*p* < 0.05).

As shown in Figure 5, the antioxidant activities of five fractions were 0.23–7.67 mg/mL, 0.08–2.11 mg/mL and 0.84–322.91 mg VCE/100 g DW for DPPH, ABTS and FRAP assays, respectively. Thus, five fractions exhibited a significant difference in antioxidant activities, and the Fr-EtOAc displayed the highest scavenging capacities of ABTS^+^ and DPPH∙ free radicals and reducing ability of Fe^3+^, followed by Fr-nBuOH and Fr-CH_2_Cl_2_, which agreed with the results of TPC and TFC (Figure 3). Similar findings had also been reported by Clodoveo et al. (50) who evaluated the antioxidant activities of phenolic compounds in sweet cherry pulp. Meanwhile, the ABTS^+^ and DPPH∙ free radicals scavenging capacities of five fractions were lower than vitamin C, but Fr-EtOAc and Fr-nBuOH exhibited excellent antioxidant activities with low IC_50_ values of DPPH (0.23, 0.38 mg/mL) and ABTS (0.08, 0.16 mg/mL) assays. Additionally, the IC_50_ value (DPPH) of Fr-EtOAc was lower than cascara (0.43 mg/mL) (51) and *Piper chaba* stem methanolic extract (0.31 mg/mL) (52). Previously, phenolic compounds could act through different antioxidant mechanisms to scavenge free radicals and reduce the high valence ions to lower valence ions (53). Furthermore, the extract of medicinal plants was also reported to remove oxidant precursors to reduce or prevent oxidative damage (54). However, the extract of the seeds of *A. villosum* was a complex mixture, and it was necessary to further explore the specific phenolic compounds that played an important role in the antioxidant activities by correlation analysis.

### 3.6 Cytotoxicity and antitumor activity

**Figure 6:**
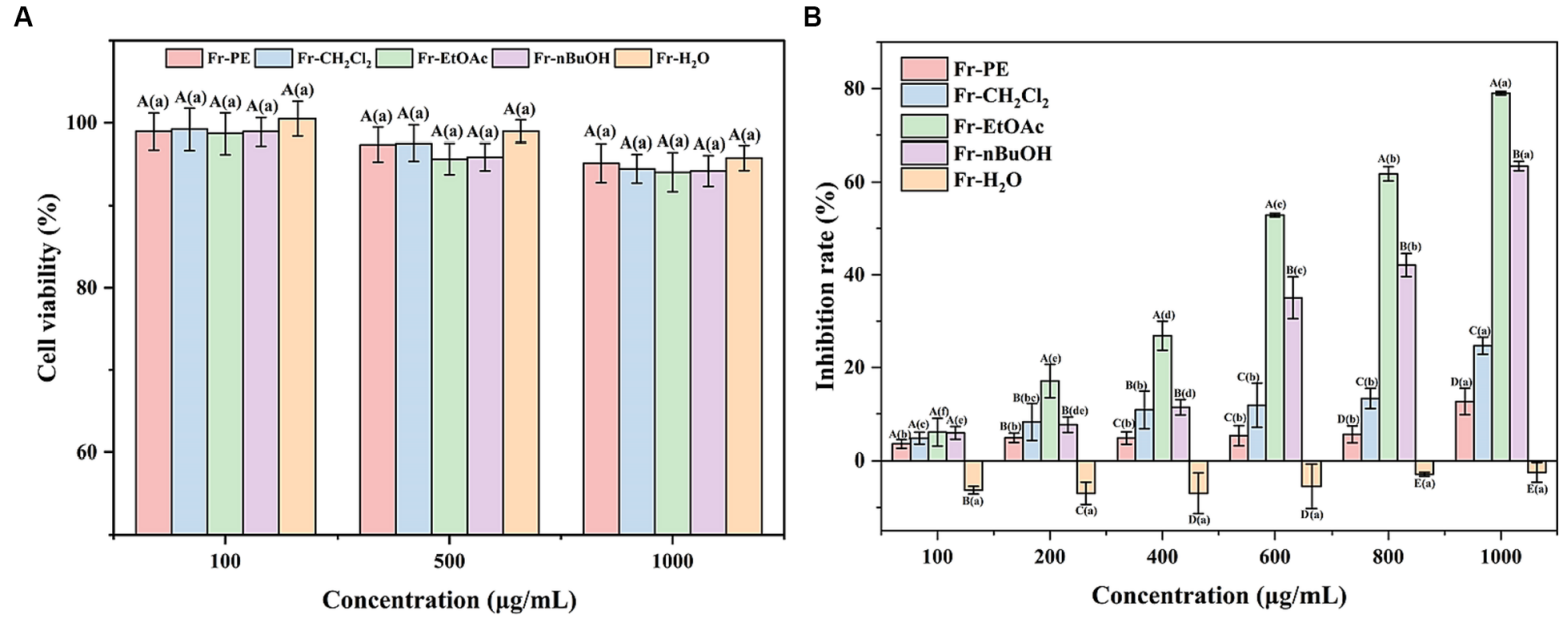
**(A)** Represented the effects of five fractions of *A. villosum* on the human umbilical vein endothelial cells viability. **(B)** Denoted the antitumor activities of five fractions of *A. villosum*. Superscript letters in each column indicated significant differences between different letters (*p* < 0.05). Fr-PE, petroleum ether fraction; Fr-CH_2_Cl_2_, dichloromethane fraction; Fr-EtOAc, ethyl acetate fraction; Fr-nBuOH, *n*-butanol fraction; Fr-H_2_O, H_2_O fraction. Capital letters represented the significant differences between different samples at the same concentration (*p* < 0.05). Lowercase letters denoted the significant differences at different concentrations of the same sample (*p* < 0.05).

*Amomum villosum*, as a medicinal and edible plant in China, can display various biological activities, including antitumor activity. Firstly, the cytotoxicity of five fractions was evaluated by measuring the cell viability of HUVE cells using a CCK8 assay. This assay is based on an analysis of the mitochondrial activity of the cells. The CCK8 assay is more sensitive than the MTT assay, which is also often used to ascertain cell viability (55). As depicted in Figure 6A, the cell viability of the HUVE cells was greater than 90% when treated with 100, 500 and 1,000 μg/mL of five fractions, respectively, indicating that these concentrations would not be considered to be cytotoxic (56). As a result, 100–1,000 μg/mL of five fractions were used in subsequent experiments because they did not promote cytotoxicity.

Subsequently, CCK8 method was further employed to evaluate the effect of five fractions on HeLa cells proliferation at concentrations of 100–1,000 μg/mL, and the results were shown in Figure 6B. The results showed that the inhibition of five fractions on the proliferation of HeLa cells was in a dose-dependent manner (100–1,000 μg/mL). Meanwhile, it was also observed that Fr-EtOAc exhibited the highest inhibition rate, followed by Fr-nBuOH, Fr-CH_2_Cl_2_, Fr-PE and Fr-H_2_O, which fitted well the trends of TPC and TFC values (Figure 3) and antioxidant activities (Figure 5). Moreover, the Fr-H_2_O had no inhibitory effect on the proliferation of HeLa cells at the test concentration (100–1,000 μg/mL).

Excitingly, in the present work, Figure 6B depicted that the inhibition rates of Fr-EtOAc and Fr-nBuOH on HeLa cells increased sharply from 6.02 to 79.04% and 5.86 to 63.45%, respectively, when the concentration increased from 100 μg/mL to 1,000 μg/mL. However, Fr-CH_2_Cl_2_ and Fr-PE displayed a slightly inhibitory effect on HeLa cells proliferation. Additionally, a previous study had also shown that ethanol extract of *Euphorbia lathyris* exhibited good antitumor activity at low concentrations (57). Therefore, the phenolic constituents of the extract may be responsible for the antitumor activity. Meanwhile, Pu et al. (58) demonstrated that ferulic acid, chlorogenic acid, caffeic acid and feruloysinapic acid in jackfruit pulp provided a significant positive contribution to the antitumor effect. Overall, the results provided foundation support for the further development of novel antitumor drugs.

### 3.7 Correlation analysis

**Figure 7:**
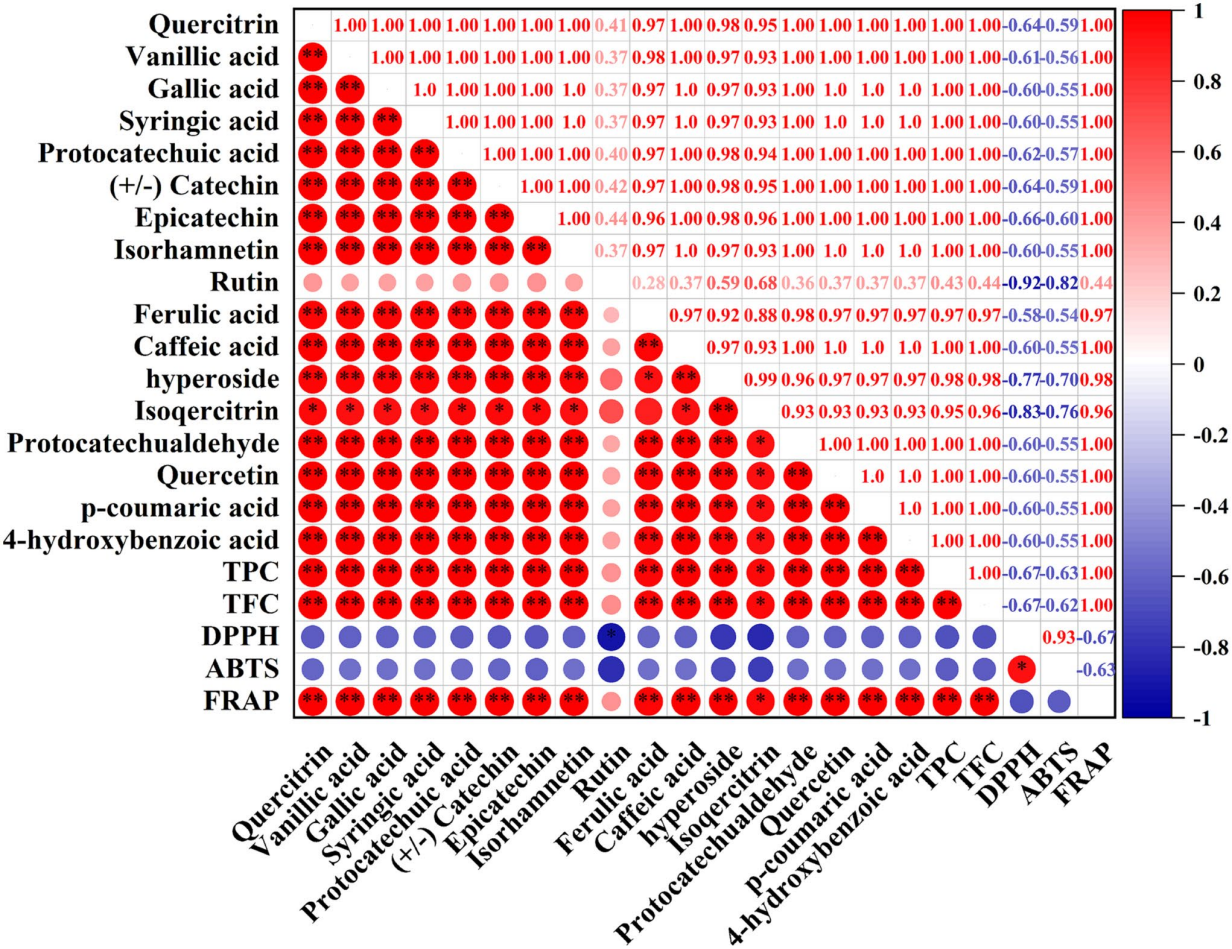
Correlation between the phenolic compounds, TPC, TFC, DPPH, ABTS, and FRAP. TPC, total phenolic content; TFC, total flavonoid content; DPPH, 1,1-Dipheyl-2-picryl-hydrazyl free radical scavenging capacity; ABTS, 2,2′-azinobis (3-ethylbenzothiazoline-6-sulphonic acid) free radical scavenging capacity; FRAP, ferric ion reducing antioxidant power. Red indicated a positive correlation, blue indicated a negative correlation, and the color intensity is proportional to the correlation coefficient. *, correlation is significant at *p* < 0.05, **, correlation is extremely significant at *p* < 0.01.

Correlation analysis has been proved to be a useful method to evaluate the correlation between individual compounds and bioactivities, which is very important to the further utilization of biological components. In this study, the correlation analysis between individual phenolic compounds and antioxidant activities (ABTS, DPPH, FRAP) was depicted in Figure 7. Intuitively, DPPH and ABTS presented a negative correlation with TPC, TFC and 17 individual phenolic compounds, while FRAP had a positive correlation with them, resulting from the results of DPPH and ABTS assays were expressed by IC_50_ values (Section 3.5). Specifically, TPC and TFC were extremely significantly (*p* < 0.01) positive correlated with FRAP and showed a negative correlation with DPPH (*r* = −0.67, −0.67, respectively) and ABTS (*r* = −0.63, −0.62, respectively). And these results also confirmed the fact that Fr-EtOAc had the highest antioxidant activities due to the highest TPC and TFC. Furthermore, it was observed that FRAP depicted extremely significant (*p* < 0.01) positive correlated with 16 individual compounds (except rutin), while DPPH was significantly (*p* < 0.05) negative correlated with rutin. Similar results were also reported by Clodoveo et al. (50) who evaluated the correlation between phenolic compounds from sweet cherry pulp and antioxidant activities (DPPH and ABTS assays). And the results of the present work demonstrated and confirmed the speculations of several recent researches on some unique phenolic compounds that contributed to higher antioxidant activities (59, 60).

## 4 Conclusion

In this work, five fractions were obtained from ethanol extract of the seeds of *A. villosum* by fractional extraction method, and the identification, quantification and biological activities (antioxidant and antitumor) of bioactive compounds in five fractions were evaluated. This study was the first report on the composition of phenolic compounds from the seeds of *A. villosum*. And the results revealed that the seeds of *A. villosum* were rich in phenolic compounds, mainly including vanillic acid, catechin, epicatechin, protocatechuic acid, quercetin and quercitrin. Meanwhile, the extract of the seeds of *A. villosum* represented excellent antioxidant and antitumor activities, which confirmed the fact that *A. villosum* had the potential to be an antitumor drug or antioxidant. However, further research is required to be conducted to better understand the antioxidant and antitumor mechanisms.

## Data availability statement

The original contributions presented in the study are included in the article/supplementary materials, further inquiries can be directed to the corresponding authors.

## Ethics statement

Ethical approval was not required for the studies on humans in accordance with the local legislation and institutional requirements because only commercially available established cell lines were used. Ethical approval was not required for the studies on animals in accordance with the local legislation and institutional requirements because only commercially available established cell lines were used.

## Author contributions

MZ: Data curation, Methodology, Writing – original draft. X-xS: Methodology, Writing – review & editing. ZW: Software, Writing – original draft. T-tD: Visualization, Writing – review & editing. C-bW: Writing-review & editing, Data curation, Investigation. YL: Validation, Writing – review & editing. J-jH: Resources, Writing – review & editing. L-qD: Conceptualization, Funding acquisition, Project administration, Writing – review & editing.
